# Stable Tin-Based Perovskite Solar Cells

**DOI:** 10.1021/acsenergylett.3c00282

**Published:** 2023-03-23

**Authors:** Antonio Abate

**Affiliations:** Department of Novel Materials and Interfaces for Photovoltaic Solar Cells, Helmholtz-Zentrum Berlin für Materialien und Energie, Kekuléstraße 5, 12489 Berlin, Germany

## Abstract

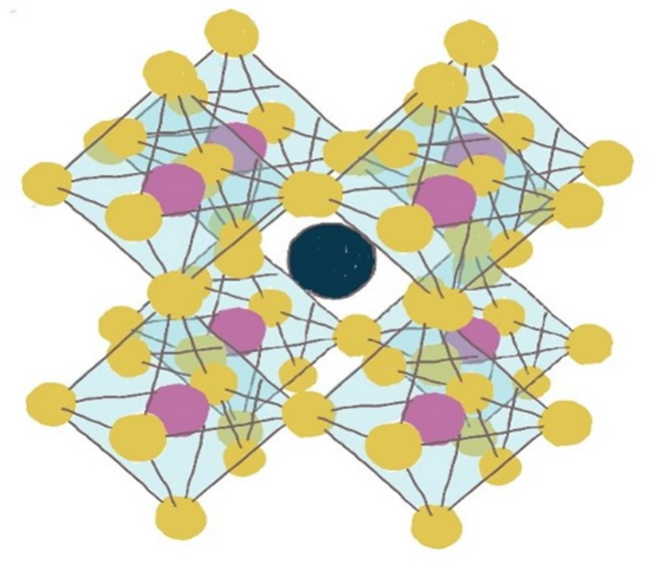

The developments in halide perovskite research target the next era of semiconductors. Photovoltaic solar cells are only one of the technologies that could be exploiting the potential of perovskites soon. Stability and toxicity are two critical aspects of photovoltaic applications because of the long-lasting lifetime and large volumes of the targeted technologies, such as multijunction solar cells with high power conversion efficiency. In this Perspective piece, I discuss how stability and toxicity can be addressed now, incentivizing the research toward lead-free and low-lead formulations. Recent works demonstrated that tin is a possible way out of the toxicity and stability issues of current perovskite formulations. I give speculative directions for stable tin-based perovskite solar cells.

Halide perovskites have been known as semiconductors for decades. They caught the scientific community’s attention shortly after Prof. Miyasaka demonstrated and followed up their application in photovoltaics, published for the first time in 2007.^1^ A few years later, this work enabled tens of thousands of scientific publications and patents that point sharply to next-generation semiconductors beyond photovoltaics, including light-emitting diodes and transistors.^2^ Traditional inorganic and innovative organic semiconductors will continue to have their market, while halide perovskites will open new and unforeseeable opportunities. One anticipated application is in multijunction photovoltaics to enable higher power conversion efficiency (PCE) at a lower cost.^3^ A perovskite multijunction photovoltaic with established materials like silicon or a solo perovskite-based device exploits two critical advantages: broadly tunable bandgap and adaptable deposition method.

Stability is the most pressing challenge in multijunction photovoltaics and many other possible applications for halide perovskites.^4^ The most stable perovskite formulations demonstrated to date are made of lead more than 30% in weight. But the use of lead can drive researchers away from investments in perovskite-based technologies because of apprehensions about lead toxicity.^5^ Tin-based perovskites have the potential to outperform the PCE and stability of lead-based perovskite solar cells.

In this Perspective piece, I will speculate on future directions for stable perovskite photovoltaics. I will discuss the most recent insights into the defect chemistry of the perovskite to overturn the common misbelief that tin creates instability. Thus, I will indicate how tin is the solution to enable stable perovskite solar cells.

Developing accelerated indoor testing to predict the stability of outdoor operation of industrially relevant prototypes is necessary for commercialization.^6^ The latest protocols run the device at the maximum power point (MPP) under constant illumination, assuming that 1k hours of MPP continuous indoor testing at room temperature is equivalent to one year of outdoor usage. Increasing the indoor testing temperature accelerates the degradation; i.e., 20+ years outdoors could be simulated in a few weeks of indoor testing.^7^

Analyzing the PCE versus the time collected at MPP under constant illumination of thousands of devices prepared in different laboratories, comprising different perovskite compositions and device architectures, I systematically observed two successive regions of time: the first few hours show a rapid change in PCE, followed by a longer-lasting quasi-steady state, as shown in Figure 1.^8^ The PCE changes collected in the first time region are entirely reversible; however, they are permanent in the second time part. In actual day–night cycling outdoor working conditions, solar cells will never reach the second time part, since they are at MPP under continuous illumination for only a few hours before resting in the dark at night. Therefore, accelerating aging under constant illumination might not predict outdoor applications well.

**Figure 1:**
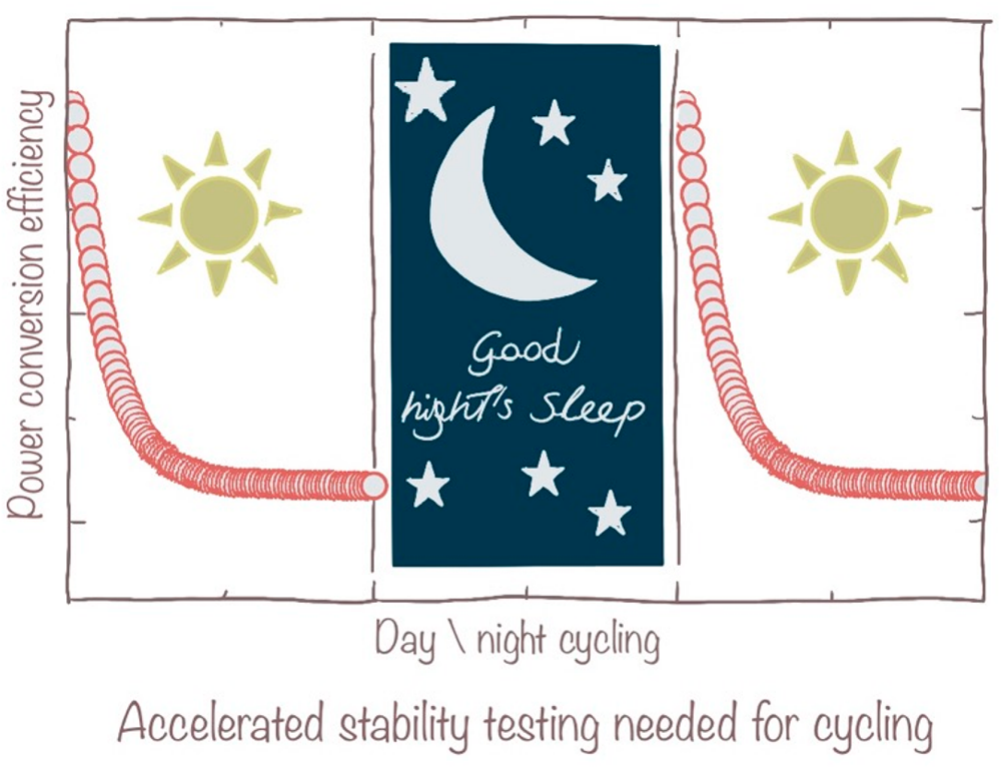
Estimating the stability of perovskite solar cells in accelerated indoor testing conditions requires cycling illumination to consider the transient behaviors in power conversion efficiency over time scales of hours, thus compatible with day–night cycling. Figure created using data from ref (8).

Cycling the illumination while keeping the device at MPP is thus necessary to design accelerated indoor testing that could predict long-term outdoor operation. Therefore, like the figure of merit used for batteries, we will talk about day–night cycles referring to the stability of perovskite solar cells. For example, 25 years of stability would require about 10k light–dark cycles at room temperature. We will simulate the lifetime of perovskite solar cells by running 10k day–night cycles, equivalent to 20 years outdoors, in a few weeks or months of accelerated lab testing by increasing the temperature and light intensity.

There are over 13k publications with “lead perovskite solar cells” in the title and only 0.3k with “tin perovskite solar cells” in the title to date, according to Clerivate Analytics. When Sn^2+^ is used to replace Pb^2+^ at the B position of the ABX_3_ perovskite lattice, a significant increase in electronic defects and the consequent doping level within the perovskite film is systematically reported and dramatically impacts the device’s PCE. A critical mechanism behind forming electronic defects is the oxidation of Sn^2+^ to Sn^4+^ during the deposition of the perovskite film.^9^

For an s^2^p^2^ element from group IV, like Pb and Sn, to form 2+ ions, it must keep the s pair and lose only the p electrons. The so-called inert s pair is more common in heavier elements such as Pb, where the relativistic contraction stabilizes the s orbitals.^10^ Accordingly, Pb is more stable in an oxidation state of 2+, while the upper group IV elements are progressively more stable in an oxidation state of 4+, i.e., Sn < Ge < Si.

Many chemicals established for processing perovskites can readily oxidize Sn^2+^ to Sn^4+^. Dimethylsulfoxide (DMSO) is a critical solvent since it strongly coordinates Sn^2+^, allowing the control of the perovskite crystallization in a homogeneous thin film. DMSO is also responsible for Sn^2+^-to-Sn^4+^ oxidation.^9,11^ Replacing or screening the impact of DMSO is necessary to prevent one of the primary sources of Sn^2+^-to-Sn^4+^ oxidation during the processing of devices. After the crystallization, residual DMSO within the perovskite film can keep oxidizing the Sn^2+^ to Sn^4+^ during the device’s lifetime.^12^

Possible strategies include replacing DMSO entirely with alternative solvents, which still enable crystallization control, or removing any residual DMSO from the perovskite film while protecting Sn during the processing.

The perovskite interface with the other materials comprising the device is another potential oxidation source that remains active throughout the device’s lifetime. This is particularly relevant for the interface with the electron contact material, which extracts electrons from the perovskite.^13^ If the Fermi level of the electron contact is significantly deeper than that of the perovskite, we might observe uncontrolled redox activity involving the oxidation of Sn^2+^ to Sn^4+^ at the interface. Metals oxide electron-selective layers, like TiO_2_ and SnO_2_, which have been used in direct contact with the perovskite, can catalyze the redox activity of Sn, thus inducing oxidation in the absence of reducing agents like oxygen, water, and residual solvent from processing like DMSO.^14^ Organic electron-contact materials can also be problematic. The commonly used C60 can act as an oxidant within the redox chemistry of tin. Isolating chemically, but not electronically, the perovskite interfaces by using an inert organic or inorganic barrier layer is one way to gain stability, as shown in Figure 2.

**Figure 2:**
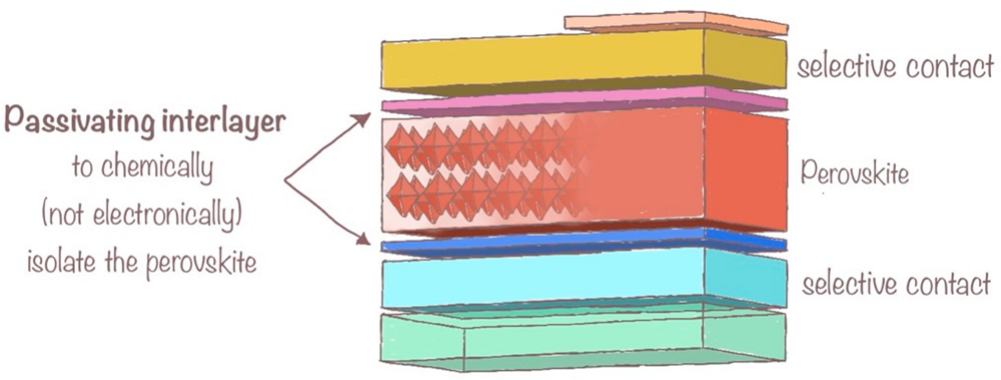
Oxidation of Sn^2+^ to Sn^4+^ is one of the primary sources of chemical and consequent electronic defects, i.e., p-doping, for tin-based perovskites. Preventing oxidation during the processing has been addressed by using reducing additives and removing unstable solvents. Still, the challenge remains to stabilize the interfaces of the perovskite layer with the other materials comprising the device. Chemical, but not electronic, barrier layers are needed to stabilize the system in working conditions.

On par with the relevance of the electronic configuration to the oxidative stability discussed in the previous section, we must consider how the inert-pair expression influences the assembly of Sn^2+^ within the perovskite structure.^10^ In Pb^2+^, the inert pair has an entirely s character, i.e., spherically distributed around the nucleus. Thus, the 6-fold coordination of lead with iodine can originate perfectly symmetric PbI_6_ octahedra, which will require assembly into a perovskite structure. Because in tin the s orbital is not significantly more stable than the p orbitals, i.e., the relativistic contraction stabilizes the s orbitals less effectively than in lead, Sn^2+^ expresses the inert pair combining the s and p orbitals in a less symmetric distribution around the nucleus, as shown in Figure 3. The inert-pair expression in Sn^2+^ can distort the SnI_6_ octahedra and the subsequent assembly into a perovskite structure. The electronic disorder will be associated with lattice distortion and negatively impact the photovoltaic performance. Yongping Fu and X.-Y. Zhu^10^ described the Sn lone-pair expression and its effect on halide perovskites in depth.

**Figure 3:**
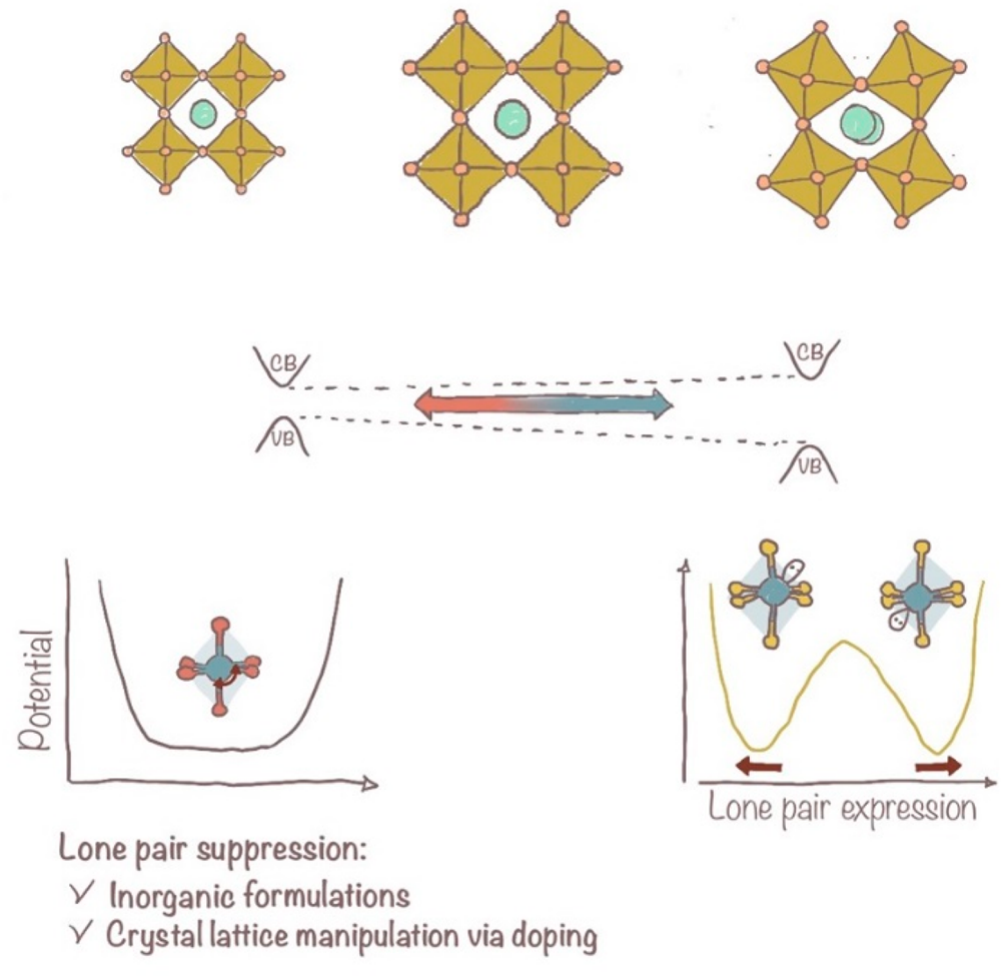
Tin lone-pair expression is a function of the formulation, and it has an important impact on the structure of the perovskite. Two possible strategies are controlling the lone-pair expression going toward inorganic formulations and including mol% quantity foreign elements within the lattice, i.e., chemical doping. Figure created using data from ref (10).

Using an inorganic cation, like cesium, at the A site of the ABX_3_ structure can reduce the lone-pair expression and, thus, the electronic disorder. Compensating the lattice distortion by doping a quantity of foreign elements within the perovskite formulation is another potential yet unexplored solution.

Under illumination and applied voltage, the migration of ionic species comprising the crystalline lattice is peculiar to halide perovskites. Indeed, halide perovskites exhibit a combination of electronic and ionic charge transport, which marks the hysteretic response of the current–voltage characteristic of perovskite solar cells. The halides, particularly the iodides, are the most mobile species within the perovskite lattice. Annamaria Petrozza and Filippo De Angelis pinpointed the core mechanism of halide migration, explaining that the oxidation of Frenkel defect interstitial iodides (I^–^) triggers a redox cascade through the generation of molecular iodine (I_2_).^15^ The formation of I_2_ in an iodide (I^–^)-rich environment enables the classical I^–^/I_3_^–^ redox chemistry, which makes the perovskite lattice resemble a solid-state electrolyte, i.e., the ionic conductivity. While the presence of mobile iodides can reduce the built-in potential and thus the maximum PCE, the redox activity of the iodides in contact with the other materials comprising the device will rapidly degrade the interfaces. The former issue, ion migration, is reversible and saturates within a few hours without destroying the device’s operation. This results in reversible performance losses under perovskite solar cells’ maximum power point tracking. Still, interface redox activity causes persistent performance degradation, which makes the device inoperational in the long run (Figure 4). Another argument supporting the use of Sn as a strategy to stabilize the perovskite is the evidence that transistors work when employing Sn while suffering an impact to ion migration in purely Pb-based formulations.^16^ Recently, direct evidence of reduced ion migration in tin-based or tin-doped perovskite has been provided.^17^

**Figure 4:**
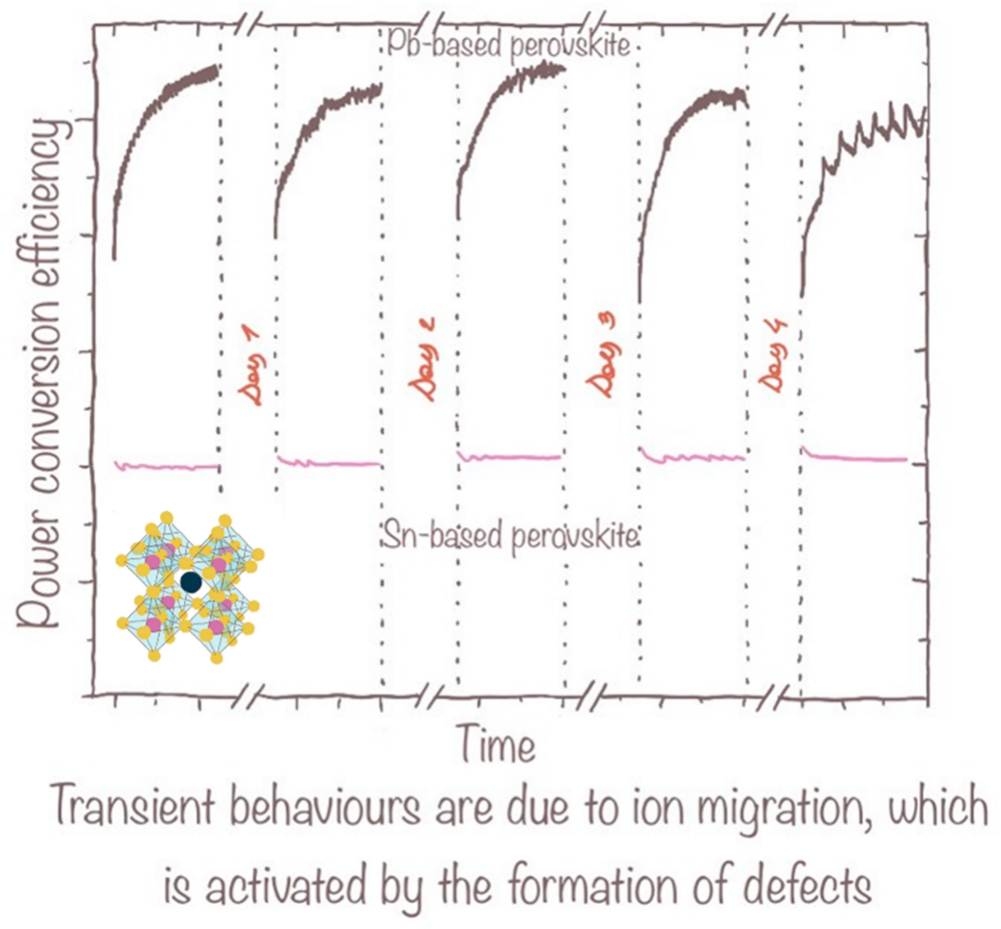
Preliminary data comparing lab-scale perovskite solar cells show that Sn lacks transient behaviors typical of Pb-based devices. This is tentatively attributed to the absence of ion migration in Sn-based perovskite devices.

The oxidation of Sn discussed in the previous section provides a significant advantage. In Pb-based perovskite, I^–^ is the easiest species to oxidize. In Sn-based perovskite, the oxidation of Sn^2+^ to Sn^4+^ has the lowest oxidation potentials, thus preventing I^–^ oxidation. The oxidation of Sn^2+^ to Sn^4+^ is preferable because the oxidation of I^–^ activates the redox chemistry of the iodine, which is detrimental to the other materials comprising the device. The lack of iodide will also reduce the occurrence of ion migration. Thus, using Sn will provide advantages for the transient performance variation on a time scale of hours (day–night cycling), the long-run stability, and the materials and interface degradation of perovskite solar cells.

I have no doubts that we are at the sunrise of a new era of semiconductors, where halide perovskites will provide opportunities unforeseeable for traditional semiconductors. Over the past few years, the research has focused more on stability and toxicity, identifying in these two aspects the remaining challenges. I speculate that Sn will offer the opportunity to tackle them both in one shot. The oxidative stability, considered a bottleneck in the use of Sn, is the chance to prepare stable and non-toxic perovskites.
